# Reformulation of Traditional Fermented *Tea* Sausage Utilizing Novel (Digital) Methods of Analysis

**DOI:** 10.3390/foods11081090

**Published:** 2022-04-11

**Authors:** Stefan Simunovic, Vesna Ž. Đorđević, Mladen Rašeta, Mirjana Lukić, José M. Lorenzo, Ilija Djekic, Igor Tomašević

**Affiliations:** 1 Institute of Meat Hygiene and Technology, Kacanskog 13, 11040 Belgrade, Serbia; vesna.djordjevic@inmes.rs (V.Ž.Đ.); mladen.raseta@inmes.rs (M.R.); mirjana.lukic@inmes.rs (M.L.); 2 Department of Animal Source Food Technology, Faculty of Agriculture, University of Belgrade, Nemanjina 6, 11080 Belgrade, Serbia; tbigor@agrif.bg.ac.rs; 3 Fundación Centro Tecnolóxico da Carne, San Cibrao das Viñas, 32900 Ourense, Spain; jmlorenzo@ceteca.net; 4 Área de Tecnología de Losalimentos, Facultad de Ciencias de Ourense, Universidad de Vigo, 32004 Ourense, Spain; 5 Department of Food Safety and Quality Management, Faculty of Agriculture, University of Belgrade, Nemanjina 6, 11080 Belgrade, Serbia; idjekic@agrif.bg.ac.rs

**Keywords:** fat reduction, fermented sausage, *tea* sausage, 3D, oral processing, computer vision

## Abstract

The main objective of this paper was to investigate the effect of fat reduction on different quality traits of *tea* sausage. This study also aimed to deploy the following digital methods of analysis: three-dimensional (3D) laser imaging, computer vision system and oral processing. Three batches of *tea* sausage with different amounts of pork back fat were manufactured: control (25%), medium fat (17.5%) and low fat (10%). Samples for the analyses were taken on the production day and after 7, 14, 21, 28 and 35 days of ripening. The fat level significantly influenced shrinkage, texture, pH, a_w_, moisture and ash contents, peroxide value, acid number, number of chewing strokes, consumption time, eating rate and fat intake rate. Oxidative stability, colour and microbiological parameters were not affected by fat reduction. The results of the sensory analysis showed that the fat level can be reduced to 17.5% without negatively affecting the quality and sensory properties of the product. The ripening time of the fat-reduced *tea* sausage should be reduced to 28 days. A strong correlation between shrinkage and weight loss suggests the possibility of using 3D laser imaging in predicting weight loss and moisture content of dry sausages.

## 1. Introduction

As products with long shelf lives and high nutritional value, fermented sausages have been produced and consumed for centuries. *Tea* sausage is a type of fast fermented pork dry sausage traditionally produced in the Western Balkan region. Its production history dates back to the beginning of the 1960s when it was introduced to consumers for the first time [1]. At the beginning, sausage was modelled after European-type fast-fermenting semidry sausages but, soon after the drying period, was extended to allowing the sausage to become dry. The *tea* sausage then became increasingly popular and, often, consumers first choice when it comes to dry sausages. It is characterized by finely comminuted meat batter, in which the consistency is achieved by the use of a bowl cutter and frozen pork back fat and meat [1]. Meat batter is stuffed in medium-diameter (38–40 mm) collagen casings and subjected to ripening for 21–35 days. The sausage contains a relatively high content of pork fatty tissue, which plays an important role in the formation of the product’s soft texture [1].

In past decades, there has been a number of papers that studied the negative effect of high fat intake on human health [2,3,4]. These usually include increased blood pressure, platelet aggregation, development of insulin resistance and diabetes, and increased risk of myocardial infarction and stroke [2,3,4]. This influenced corporate social responsibility (CSR) activities from food companies to become more focused on nutrition initiatives, especially on the development of low-fat products [5,6]. However, low-fat products are usually found to be of inferior quality from the consumer point of view [7] as fat affects flavour, juiciness, appearance and texture of food. Although there is a number of fat-reduced products available on the market, there is a lack of low-fat meat products, especially fermented sausages. Reducing the fat content from a sausage formulation is not a trivial task [8] because, apart from its effect on sensory attributes, fat is also very important from a technological point of view due to its positive influence on the continuous release of moisture from the inner layers of the sausage [9].

In the production of fermented sausages, there has been two main approaches to reducing the negative health impact of dietary fat. The first implies a reduction in the amount of animal fat used in sausage formulations and its replacement with various types vegetable oils (olive, grapeseed, linseed, etc.) in the form of different gels. This is because vegetable oils are considered a healthy alternative to animal fats as they contain lower levels of saturated fatty acids (SFA) and lower *n*-6/*n*-3 ratio. However, these products, in most cases, failed to meet consumer demands in terms of flavour and were more susceptible to oxidation due to their higher content of unsaturated fatty acids [8,9,10]. The second approach includes a direct reduction in fatty tissue content in sausage formulations by increasing the content of lean meat [11]. The main disadvantages of this approach is the formation of an intensively wrinkled surface of the sausage and higher production costs as a result of the higher content of meat used in the formulation [9]. The latter point is probably the reason why the meat sector avoids reducing the fat content at the expense of increasing lean meat content in its products. Nevertheless, the recent study of Cullere et al. (2020) showed that a sausage formulated with a lower fat level had the highest initial moisture content and significantly lower moisture content at the end of ripening than that found in sausages formulated with higher levels of fat. Increased drying kinetics of low-fat sausages suggest the possibility of reducing their drying time and therefore improving the cost-effectiveness of their production.

This study aims to demonstrate the possibility of formulating a low-fat and healthier type of tea sausage and to investigate the effect of fat reduction on different quality traits of *tea* sausage with reduced ripening time. In addition, a survey was conducted in order to find out consumer attitudes towards fat content in fermented sausages and their behaviours at the point of purchase. The novelty of this research lies within the application of a number of novel digital analytical methods such as three-dimensional (3D) laser imaging, computer vision system (CVS) and oral processing.

## 2. Materials and Methods

### 2.1. Survey

An online survey, in which 854 citizens of Serbia took part, was conducted prior to defining experiment design. The survey was organized into three following parts: (1) socioeconomic demographic characteristics, (2) consumer’s behaviour at a point of dry sausage purchase and consumption frequency, and (3) consumer’s attitudes towards fat content in fermented sausages. The minimum sample size for the survey was found to be 400 and was calculated according to Israel [12], with a confidence level of 95%. Only fully completed questionnaires were analysed.

### 2.2. Sausage Production

Three independent batches of *tea* sausages (40 kg each) with different amounts of pork back fat (25%, 17.5% and 10%) were manufactured: high fat (HF) (control), medium fat (MF) and low fat (LF). The rest of the meat batters consisted of lean pork leg. Back fat and one part of the pork leg were frozen (−18 °C) and cut in bowl cutter KU 130 AC (Laska, Traun, Austria) prior to the addition of previously minced pork leg (4 mm diameter plate), salt (2.2%), white pepper (0.15%), black pepper (0.15%), garlic (0.2%), dextrose (0.6%), sodium nitrite (110 mg/kg) and starter culture mixture cT salami fast (CreaTec GmbH, Friedrichshafen, Germany). The batter was mixed for 2 min and stuffed into 40 mm diameter collagen casings using vacuum filler VF616 (Handtmann, Biberachan der Riss, Germany), making around one hundred and fifty sausages of approximately 20 cm in length. After a rest day, the sausages were transferred to a drying/ripening chamber, where they were cold smoked for 3 days using beech chips and kept until the end of the ripening. The temperature and relative humidity (RH) conditions in the ripening chamber were the following: 1–3 days (23 °C, 90–95% RH), 4–6 days (20 °C, 85% RH), 7–9 days (18 °C, 80% RH) and 10–35 days (12–15 °C, 70% RH). Samples for the analyses were taken after stuffing them in the casings and after 7, 14, 21, 28 and 35 days of production. All samples were transferred to a laboratory at 4 °C.

### 2.3. 3D Laser Imaging

The sausages were scanned using EinScan-SP (Shining 3D Tech., Hangzhou, China) 3D scanner. The accuracy of the scanner was 0.03 mm, with a point distance between 0.17 and 0.20 mm and a scan speed < 1 min. The resolution of the two cameras was 1.3 Mega Pixels, while an integrated white LED light was used for lighting of the samples. Prior to each sampling stage, the scanner was calibrated using the calibration board provided by the manufacturer. The samples were placed on a turntable, scanned and then rotated for 90° in order to obtain data for the top and bottom surfaces of the sausage. Similar to in the study of Vaskoska et al. [13], scanning of each sausage resulted in the formation of approximately 9 million point clouds. The sausages were scanned using the turntable align mode, while the number of turntable steps was set to 50 for each of two positions. The total scanning time was around 40 min per sample. The scanner was connected to an upgraded Lenovo Legion (Windows 10, intel core i5-73000 HQ CPU, 2.5 GHz, 24 GB RAM memory, GeForce GTX 1050 2 GB graphic card and solid-state drive 500 GB) PC. The point clouds were meshed using EXScan S_v3.0.0.1 (Shining 3D Tech., Hangzhou, China) software using high-detail and watertight meshing modes, as proposed by Wong et al. [14]. Prior to meshing, all background details were trimmed off. The relative error of the 3D scanned volume at each analysis stage was calculated by comparing the 3D volume with the volume obtained using the water displacement method on random samples of the tea sausages, as recommended by Zhang et al. [15]. To not affect the drying of the sausages, a 3D analysis was performed at the production plant and the sausages were returned immediately after scanning.

### 2.4. CVS Analysis

The lightness (L*), redness (a*) and yellowness (b*) of the *tea* sausage casing, meat and fat parts were measured independently using CVS according to Tomašević et al. [16] with some modifications. First, the camera was calibrated using X-Rite Colorchecker Passport (Grand Rapids, Michigan, MI, USA), after which the surface of the sausage and the casing were photographed. Instead of 2 cm thick slices, as suggested in the study of Tomašević et al. [16], the sausages were cut into 1 cm thick slices and placed on white paper. Images of the sausage cross sections were later used to obtain the colour parameters of both the meat and fat parts. The time between cutting the sausage and taking the photo was less than 30 s.

### 2.5. TPA Analysis

TPA was performed as described by Simunovic et al. [17], with some modifications. The sausages were cut to 10 mm thick slices for which the weight and diameter were measured prior to compression. The values obtained for each slice separately were entered into TEE32 Exponent 4.0.12. (Stable Micro Systems Ltd., Vienna Court, Godalming, UK) software. The following parameters were obtained: hardness (*N*), springiness, cohesiveness, gumminess (*N*), chewiness (*N*) and resilience.

### 2.6. Physicochemical Analyses

The determination of pH, water activity (a_w_), total protein, fat, moisture and thiobarbituric acid-reactive substances (TBARS) content was performed as previously described in our previous study [17]. As an additional indicator of lipid oxidation, peroxide and acid value were analysed in accordance with ISO 3960:2017 and ISO 660:2020, respectively.

### 2.7. Microbiological Analyses

Ten grams of each sample were weighted and transferred to sterile plastic bags, in which 90 mL of Buffered Peptone Water (BPW) (Oxoid, Basingstoke, UK) was added. Total viable counts (TVC) and lactic acid bacteria (LAB) were determined according to the ISO 4833-1:2013 and ISO 15214:1998 methods. At each stage, the analyses were performed in triplicate.

### 2.8. Descriptive Sensory Analysis

The sensory panel consisted of seven male and five female members, each well-experienced in analysis of meat products. The sensory panel was trained for two months in accordance with the suggestion by Djekic et al. [18]. During the introductory session, the panel defined and agreed upon fourteen sensory traits, which were rated on 10-point Likert scale. The selected parameters were the following: fattiness, fat/meat cohesiveness, fat distribution, meatiness, hardness, juiciness, dryness, elasticity, sourness, saltiness, black pepper, smoke, lactic acid and garlic flavour. In addition, the panel evaluated the following attributes of sausages at the end of the ripening using a five-point Likert scale: colour, aroma, taste, consistency and overall acceptability. One part of the sausages from each batch was vacuum packed after 28 days of ripening and stored at 4 °C while the other part was packed after 35 days of ripening. This was performed in order for the sausages to be evaluated at the same time.

### 2.9. Oral Processing

Prior to analysis, the samples were cut into 5 mm thick slices. Each of the eight panellists received three identical slices, from each of the batches, for which the total weight was measured before and after mastication using a technical balance. The panellists were recorded using a digital video camera during analysis while two researchers analysed the videos and, afterwards, compared the results. The oral parameters obtained were in accordance with the study of Djekic et al. [19], with some modifications: number of chews, consumption time of one bite (s), chewing rate (chew/s), average bite size (g) and eating rate (g/s). Fat intake rate (fat/s) was calculated by multiplying the eating rate with the percentage of average fat content in each sample.

### 2.10. Statistical Analysis

Statistical analysis was performed using SPSS (SPSS 23.0, Chicago, IL, USA) software. The correlation between variables was determined by the Pearson’s linear correlation coefficient, while the difference between mean values were tested using one-way ANOVA and Tukey’s post hoc test (*p* < 0.01). The normality of the data and the homogeneity of variance were assessed by Levene and Shapiro–Wilk tests. The results of the survey and descriptive sensory analysis were processed by MS Excel (Microsoft, Redmond, WA, USA).

## 3. Results and Discussion

### 3.1. Survey

The sample consisted of 54.8% female and 45.2% male participants, and 63.7% of them were under the age of 40, with a median between 30–39 years of age. The majority of the participants had faculty degrees (53.2%), and the rest had high school degrees (35.2%), were students (9.8%), or had primary-school-level education (1.8%). Most of the respondents were employed (80.3%), followed by unemployed (16.9%), while 2.8% of them were retired. The highest number of them were from Belgrade (71.2%), followed by Šumadija and Western Serbia (12.2%), Southern and Eastern Serbia (9.6%), and Vojvodina (7.0%). The results of the survey revealed that the majority of respondents (41.9%) consume dry sausages once a week, while 29.9% and 26.8% of them consume sausages once every two to three days or once a month, respectively. When it comes to the consumption of dry sausages per month, the most frequent answers were less than 200 g (42.4%), between 200 and 500 g (37.2%), and between 500 and 1000 g (13.8%). The popularity of *tea* sausage among consumers in Serbia was confirmed by the survey as 29.5% participants identified *tea* sausage as their first choice regarding dry sausages, while 28.1% and 19.7% identified it as the second and the third choice, respectively. The majority of participants (82.7%) believe that excessive fat intake causes health problems in humans, while 63.0% found it important that *tea* sausage contains as little fat as possible. When it comes to the appearance of the sausage, 58.08% of consumers do not find it important if the surface of the sausage is wrinkled. On the other hand, opinions are divided as to whether they negatively perceive if the sausage is harder than usual. However, the majority of consumers (77.99%) are willing to pay more for sausages containing less fat (more meat).

### 3.2. 3D Laser Imaging

**Figure 1:**
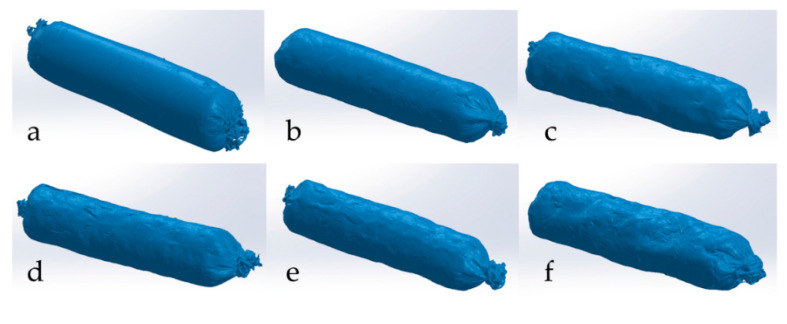
Three-dimensional (3D) models of low-fat *tea* sausage (MF) during dying: (**a**) production day, (**b**) 7th day, (**c**) 14th day, (**d**) 21st day, (**e**) 28th day and (**f**) 35th day.

In all analysed batches of *tea* sausage, the 3D estimated volume significantly (*p* < 0.01) decreased during 35 days of ripening (Figure 1) (Table 1 and Table 2). The relative error (%) of the 3D estimated volume was found to between 2.4% and 5.2%, which is in agreement with those reported by other authors [15,20]. The fat level showed a significant (*p* < 0.01) effect on the shrinkage of the sausage during all stages of ripening. The highest shrinkage was observed for LF sausages, which reduced to 44.4% of their initial volume at the end of ripening. On the other hand, MF and HF sausages showed significantly (*p* < 0.01) lower shrinkage, reducing to 39% and 29% of their initial weights, respectively. It is important to mention that significant (*p* < 0.01) differences in shrink age among the batches were observed after the first seven days of ripening. Shrinkage showed a very strong correlation (*p* < 0.01) with weight loss (r = 0.99), suggesting the possibility of using 3D laser imaging in weight loss prediction. In recent years, there has been a number of studies on the application of 3D printing and scanning technology in food processing and analysis [15,20,21,22]. The latter can be used to generate databases of various of food products that would be used in laboratories globally. These databases could possibly enhance geometrical characterization and analysis of products that are, to some extent, unknown to a certain laboratory. However, this still presents an unknown field to researchers and it is a first step in development of 3D laser food imaging analytical solutions in the future [23].

### 3.3. CVS

Recently, Tomašević et al. [16] proposed a CVS-based method for measuring the colour of meat and meat products. The main advantage of this method is the possibility of measuring the colour of meat and fat parts of bicoloured meat products, such as fermented sausages, separately. Hence, in this study, the L*, a* and b* values for meat and fat were measured independently (Table 1 and Table 2). Fat level did not affect the colour of the surface, or the meat and fat parts of *tea* sausage. Our results are in disagreement with the results reported by a number of authors for different dry sausages [11,24,25,26]. In these studies, it was reported that, by increasing the fat level, the lightness of sausages increases. This may be because colour in these studies was measured using a traditional colourimeter, which measures the average colour of an area on top of which the aperture size is placed. As the cross section of fermented sausages is formed by evenly distributed pieces of meat and fat, colourimeters are not able to differentiate these two phases. Hence, in the case of sausages formulated with higher fat levels, there is more fatty tissue on the cross section and this may be the reason why higher values of lightness were obtained in these studies. On the other hand, the colour parameters of *tea* sausages were significantly (*p* < 0.01) affected by ripening time, which is in accordance with the results of other authors for different types of dry sausages [11,17,26,27]. In the study by Altmann et al. [28], it was shown that consumers are able to notice very small changes in the colour of meat. The L*, a* and b* values of meat and fat parts of *tea* sausage obtain were similar to those reported in our previous study for Njeguška [17] and to those reported by Tomašević et al. [16] for fermented pork sausage.

### 3.4. TPA

The results obtained by TPA analysis are presented in Table 1. The fat level showed a significant (*p* < 0.01) effect on the texture parameters of *tea* sausages. The hardness of the sausage was significantly (*p* < 0.01) lower in the case of HF sausages compared with MF and LF sausages during the entirety of ripening. Generally, the higher the fat level is, the lower the values of hardness were. These results are in accordance with those reported in the study by Lorenzo and Franco [11], who evaluated the effect of fat level on physicochemical traits of dry foal sausage. By decreasing the fat level, the values of gumminess and chewiness significantly (*p* < 0.01) increased. In contrast, cohesiveness and resilience were found to be significantly higher in LF batch sausages than those found in HF and MF batches. However, no significant (*p* < 0.01) differences in cohesiveness and resilience were observed between sausages of the HF and MF batches. This is in disagreement with the results of Lorenzo and Franco [11], who found no significant (*p* < 0.001) differences in the cohesiveness of foal sausages produced with different fat levels. Moreover, ripening time significantly (*p* < 0.01) affected the increase in hardness, gumminess and chewiness which was also observed by other authors for different types of fermented sausages [11,17,27].

### 3.5. Physicochemical Analysis

Fat level showed a significant (*p* < 0.01) effect on the pH value of *tea* sausages, which was found to be the lowest in HF sausages, followed by the MF and LF batches (Table 1 and Table 2). This may be because an a_w_ < 0.90 value was achieved later in the case of sausages with higher fat contents (HF and MF). More precisely, the a_w_ measured on the 14th day of ripening in HF, MF and LF sausages was 0.915, 0.901 and 0.894. Therefore, LAB and other acid-producing bacteria were active for a longer period of time in the case of sausages formulated with a higher fat level. On the other hand, the results showed that, the higher the fat level, the lower the protein content and the higher the fat content. As sausages were produced with different levels of fatty tissue, the initial moisture content was significantly (*p* < 0.01) different among the batches, with the highest being in LF sausages. This is because meat contains more moisture than fat, which was observed in a number of studies [9,25,26,29]. However, at the end of the ripening, LF sausages contained the lowest moisture content as a result of the more intense drying. The moisture content in MF and LF sausages dropped under national regulation limit (35%) after 28 days of drying, which means that from a legal point of view, the drying time can be reduced by seven days. On the other hand, the moisture content of HF sausages, which were used as control, went below 35% after 35 days of ripening. Moisture content was strongly related (*p* < 0.01) to weight loss (r = 0.99). Furthermore, sausages produced with a reduced fat content showed significantly (*p* < 0.01) higher levels of weight loss during ripening. These results are in accordance with the results of Cullere et al. [30] and Muguerza et al. [9], who evaluated the effect of fat reduction on different quality traits of salami. At the end of the ripening, the ash content was found to be significantly higher in LF sausages. This was also observed by Muguerza et al. [9], who investigated the reduction in fat and its replacement by olive oil in dry sausages.

### 3.6. Oxidative Stability

The primary products of lipid oxidation were monitored by determining peroxide value as an indicator of the formation of peroxide and hydroperoxide groups. At the end of ripening, the peroxide values were found to be the significantly (*p* < 0.01) different among all batches, with the highest being in sausages of the HF batch, followed by MF and LF (Table 2). Hydroperoxides are generally considered odourless and tasteless and, because of that, do not affect product quality. However, they are very unstable and react quickly to form secondary products of lipid oxidation that include aldehydes, ketones, alcohols and epoxides, which are directly responsible for the deterioration of sensory properties in the product [31]. Lipid oxidation is the most common quality defect when it comes to processed meat since it results in the development of rancid flavour and changes in the colour of the product. The level of secondary products of lipid oxidation was evaluated by determining TBARS, which remained at relatively low levels during the entirety of ripening. The fat level showed no significant (*p* < 0.01) effect on TBARS. Our results are in agreement with those reported by Bolumar et al. [24], who observed no significant differences between TBARS found for sausages formulated with different fat levels. On the other hand, fat level significantly (*p* < 0.01) affected acid number, which was found to increase with a reducing fat content. According to the ISO 660:2009 method, acid number can be expressed as an estimated percentage of free fatty acids. Hence, LF sausages showed the highest free fatty acid levels. However, the method is unable to differentiate between mineral acids, free fatty acids and other organic acids.

### 3.7. Microbiological Analysis

**Figure 2:**
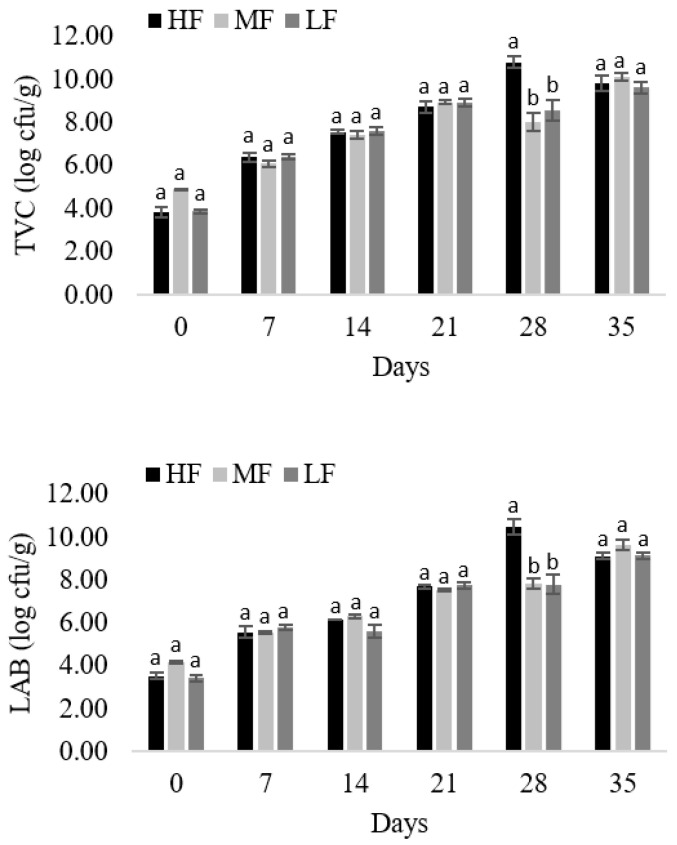
Total viable count (TVC) and lactic acid bacteria (LAB) counts in *tea* sausage produced with reduced fat content. ^a^^–^^b^ Mean values followed by different letter differ significantly (*p* < 0.01).

Changes in the counts of TVC and LAB are presented in Figure 2. The fat level did not show a significant (*p* < 0.01) effect on the microbial counts during ripening. The initial TVC counts ranged from 3.86 to 4.91 log cfu/g and significantly (*p* < 0.01) increased over the course of ripening to around 9.90 log cfu/g. Similarly, ripening time showed a significant effect on LAB counts, which gradually increased from around 3.80 log cfu/g to 9.35 log cfu/g at the end of the ripening. Both the initial and final TVC and LAB counts were similar to those reported by different authors for various types of dry sausages [11,17,27]. After 28 days of ripening, significantly (*p* < 0.01) higher TVC counts were observed for HF sausages. This can be explained by the fact that LAB, which counts were also elevated for HF batches, is the most dominant flora in TVC. However, at the end of the production, no significant (*p* < 0.01) differences between TVC and LAB counts were observed among batches. An increase in microbial counts was most pronounced during the first seven days of ripening, when the conditions for their reproduction were optimal. This was also observed in other types of dry sausages by a number of authors [11,17,32,33,34].

### 3.8. Descriptive Sensory Analysis

The sensory evaluation conducted by the experienced sensory panel was conducted on sausages of each batch that were ripened for 28 and 35 days, making a total of six different samples (Table 3). When it comes to HF (control) sausages, higher scores were obtained for sausages that ripened for 35 days, as expected. On the other hand, MF and LF sausages that were ripened for only 28 days obtained higher values than those that were ripened for 35 days. However, the overall acceptability of all sausages except MF sausages that were ripened for 28 days were found to be significantly (*p* < 0.01) lower than those obtained for control sausages after 35 day of ripening. Low-fat products are usually found to be of inferior quality from the consumer point of view [7] as fat affects flavour, juiciness, appearance and texture of food. In addition to the role of fat in terms of sensory properties, fat is also considered very important from the technological point of view due to its positive influence on the continuous release of moisture from the inner layers of the sausage [9].

**Figure 3:**
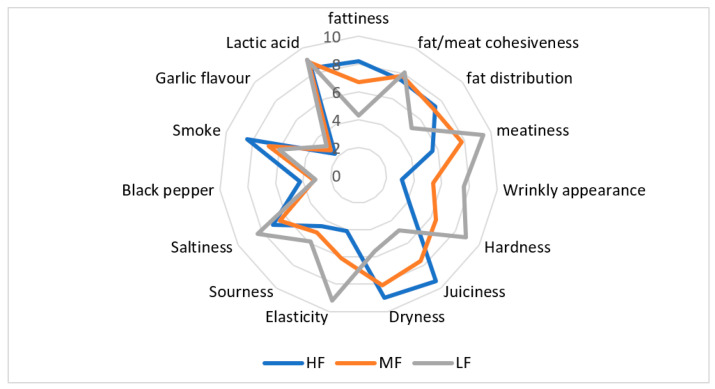
Results of descriptive sensory analysis of *tea* sausage with reduced fat content.

*Tea* sausage is characterized by intense acidic flavour as a result of fermentation mainly by LAB. This is in accordance with the relatively low pH value obtained for all analysed batches. The results of the descriptive sensory analysis showed significant (*p* < 0.01) differences in terms of fattiness, meatiness, wrinkly appearance, hardness, juiciness, dryness and elasticity between the batches of tea sausage analysed (Figure 3). On the other hand, fat level did not affect fat/meat cohesiveness, sourness, saltiness, black paper, smoke, garlic and lactic acid flavour. The highest scores for wrinkly appearance were obtained for LF sausages, which maybe one of the reasons for its lowest values in overall acceptability. The wrinkly appearance is a result of weight loss, which was the highest in LF sausages. This is in accordance with the study by Wirth [35], who found that a high fat content slows drying rate during ripening, which results in lower amount of wrinkles compared with sausages produced with a lower fat content. In addition, the longer the ripening period, the higher weight loss is and, consequently, the wrinklier a sausage appears.

### 3.9. Oral Processing

The best-rated sausages from each beach, in accordance with the results of sensory evaluation (Table 3), were selected and subjected to oral processing analysis. The chosen HF sausages were those that were ripened for 35 days, while for the MF and LF batches, sausages that were ripened for 28 days were selected. The results of the oral processing analysis are presented in Table 4. Although the number of chewing strokes differed between the batches, no significant (*p* < 0.01) difference was observed as a result of the relatively high standard deviation. High values of standard deviation and standard error were reported in the number of studies in which oral processing analyses were conducted [19,36,37]. The consumption time of one bite was found to be similar for sausages of the HF and MF batches but significantly (*p* < 0.01) higher in the case of LF sausages. Our results are in accordance with those of Peyron et al. [37], who found that, by increasing hardness of sample, the number of chewing strokes and consumption time increased. Furthermore, the chewing rate (chew/s) observed for HF and MF sausages was found to be similar but higher than that of LF sausages. This could be explained by the increased hardness of LF sausages, which requires greater masseter muscle activity and, consequently, results in slower chewing. Similar results to those found in our study were observed by Peyron et al. [37], who reported a slight decrease in chewing rate for the hardest sample analysed. On the other hand, the average bite size between the batches was found to be similar among the batches due to uniform cutting of the samples prior to analysis by researchers. Fat reduction significantly affected eating rate (g/s), which was found to be significantly (*p* < 0.01) lower in LF sausages compared with those found for HF sausages. However, the eating rate found for MF sausages was similar to those obtained for both LF and HF sausages. The lower values observed for the LF batch could be explained by the longer consumption time, which is used in the calculation of eating rate. In addition, fat level significantly (*p* < 0.01) affected fat intake rate (fat/s), which was the highest in the case of HF sausages, followed by sausages of the MF and LF batches.

## 4. Conclusions

The content of pork back fat in the formulation of *tea* sausage can be reduced to 17.5% without negatively affecting its quality and sensory properties when the ripening time is reduced to 28 days. A reduction in fat resulted in an increase in shrinkage of the sausage, which was found to be strongly related with weight loss suggesting the possibility of predicting weight loss and moisture content by means of 3D laser imaging in future. Texture, pH, a_w_, ash contents, peroxide value, acid number, number of chewing strokes, consumption time and eating rate were all affected by a reduction in pork back fat in sausage formulations. On the other hand, fat reduction did not compromise the oxidative stability, colour and microbiological parameters of tea sausage.

The results of the survey revealed that consumers would rather buy *tea* sausage with reduced fat levels and that they are willing to pay more for it even though this sausage contains more wrinkles. A reduction in ripening time from 35 to 28 days of fat reduced sausages (HF and MF) resulted in higher scores in sensory attributes and overall acceptability. In addition, digital methods of analysis were successfully deployed and are recommend in future analyses of quality properties of meat products. Although the formulation of low-fat sausages is more expensive, companies can, to a certain extent, compensate for this increase in production costs by reducing the ripening time by seven days. Fat intake rate (fat/s) decreases by decreasing the fat level, which can help consumers achieve their health goals regarding reductions in total fat intake. This is particularly important as the survey results revealed the popularity of *tea* sausage in the Western Balkans.

## Figures and Tables

**Table 1:** Changes in geometrical, physicochemical, oxidative, textural and colour parameters of *tea* sausage produced with reduced fat content in the first fourteen days of ripening.

Processing Time (Days)													
		0			SEM	7			SEM	14			SEM
		HF	MF	LF		HF	MF	LF		HF	MF	LF	
Shrinkage (%)						17.71 ^1a^	22.51 ^2a^	23.55 ^3a^		22.66 ^1b^	30.74 ^2b^	35.63 ^3b^	1.92
Chemical parameters													
pH		5.39 ^1a^	5.39 ^1a^	5.40 ^1a^	0.00	4.95 ^1b^	5.05 ^2b^	5.13 ^3b^	0.03	4.93 ^1b^	4.99 ^2c^	5.11 ^3c^	0.03
a_w_		0.939 ^1a^	0.943 ^1a^	0.943 ^1a^	0.00	0.931 ^1b^	0.930 ^1b^	0.932 ^1b^	0.00	0.915 ^1c^	0.901 ^2c^	0.894 ^3c^	0.00
Protein (% DM)		33.14 ^1a^	39.70 ^2a^	46.17 ^3a^	1.89	34.15 ^1a^	39.13 ^2a^	46.10 ^3a^	1.74	33.91 ^1a^	39.00 ^2a^	45.27 ^3a^	1.65
Fat (% DM)		59.00 ^1a^	55.59 ^2a^	44.46 ^3a^	2.20	56.60 ^1b^	52.61 ^2b^	43.48 ^3ab^	1.95	58.98 ^1a^	54.00 ^2ab^	45.82 ^3a^	1.92
Moisture (%)		54.94 ^1a^	58.40 ^2a^	61.93 ^3a^	1.01	45.09 ^1b^	50.35 ^2b^	53.97 ^3b^	1.29	42.48 ^1 c^	41.73 ^2c^	40.27 ^3c^	0.32
Ash (%)		3.02 ^12a^	2.95 ^1a^	3.16 ^2a^	0.03	3.54 ^1b^	3.71 ^2b^	3.78 ^2b^	0.04	3.66 ^1b^	4.16 ^2c^	4.83 ^3c^	0.17
Weight loss (%)						16.51^1a^	22.84 ^2a^	26.04 ^3a^		22.34 ^1b^	32.08 ^2b^	37.43 ^3b^	2.21
Lipid oxidation													
TBARS (mg MAL/kg)		0.04 ^1a^	0.03 ^1a^	0.04 ^1a^	0.00	0.04 ^1ac^	0.05 ^1ab^	0.06 ^1ab^	0.00	0.05 ^1abc^	0.06 ^1ab^	0.07 ^1ab^	0.00
Acid value (mg KOH/g)		0.93 ^1a^	1.55 ^2a^	1.99 ^3a^	0.15	1.00 ^1a^	1.65 ^2a^	1.66 ^2b^	0.11	2.32 ^1b^	3.47 ^2b^	4.33 ^3c^	0.29
Peroxide value (meq/kg)		4.24 ^1a^	4.68 ^2a^	4.14 ^1a^	0.08	4.30 ^1a^	4.74 ^2a^	3.21 ^3b^	0.23	3.54 ^1b^	1.52 ^2b^	0.90 ^3c^	0.40
TPA test													
Hardness (*n*)		5.00 ^1a^	7.12 ^2a^	8.97 ^3a^	0.34	13.01 ^1b^	23.38 ^2b^	36.44 ^3b^	1.91	24.12 ^1c^	38.70 ^2c^	50.57 ^3c^	2.28
Springiness		0.62 ^1a^	0.63 ^1a^	0.71 ^2a^	0.01	0.64 ^1a^	0.73 ^2b^	0.81 ^3b^	0.02	0.60 ^1a^	0.70 ^12bc^	0.69 ^2a^	0.01
Cohesiveness		0.42 ^1a^	0.47 ^2ab^	0.43 ^12a^	0.01	0.39 ^1a^	0.45 ^1a^	0.53 ^2b^	0.01	0.46 ^1ab^	0.49 ^2ab^	0.52 ^2bc^	0.01
Gumminess (*n*)		2.06 ^1a^	3.13 ^2a^	3.77 ^3a^	0.15	6.99 ^1b^	10.41 ^2b^	15.87 ^3b^	0.74	12.48 ^1c^	20.87 ^2c^	30.19 ^3c^	1.46
Chewiness (*n*)		1.31 ^1a^	2.03 ^2a^	2.61 ^3a^	0.11	4.72 ^1b^	7.12 ^2b^	12.87 ^3b^	0.70	7.22 ^1b^	12.68 ^2c^	20.15 ^3c^	1.08
Resilience		0.06 ^1a^	0.09 ^2a^	0.08 ^2a^	0.00	0.08 ^1ab^	0.10 ^2abc^	0.17 ^3b^	0.01	0.10 ^1bc^	0.12 ^2b^	0.13 ^2c^	0.00
Colour													
Meat	L*	51.43 ^1a^	51.57 ^1a^	51.29 ^1a^	0.66	49.86 ^1ab^	51.71 ^1a^	51.29 ^1a^	1.47	43.71 ^1b^	43.43 ^1b^	40.57 ^1a^	0.87
	a*	39.14 ^1a^	38.57 ^1a^	39.43 ^1ac^	0.63	35.86 ^1ab^	35.43 ^1ab^	35.71 ^1abc^	0.54	33.86 ^1ab^	32.71 ^1b^	33.43 ^1bc^	0.61
	b*	15.71 ^1a^	16.14 ^1a^	16.86 ^1a^	0.57	13.57 ^1a^	12.14 ^1ab^	14.14 ^1ab^	0.60	13.71 ^1a^	12.57 ^1ab^	13.85 ^1ab^	0.63
Fat	L*	81.57 ^1a^	79.71 ^1ab^	81.71 ^1a^	0.51	78.57 ^1a^	82.14 ^1b^	81.71 ^1a^	0.60	70.57 ^1b^	68.86 ^1c^	71.29 ^1b^	0.94
	a*	8.57 ^1ab^	9.86 ^1a^	7.86 ^1abc^	0.41	9.57 ^1ab^	7.86 ^1ab^	8.14 ^1abc^	0.38	12.86 ^1a^	11.29 ^1a^	9.71 ^1a^	0.68
	b*	1.14 ^1a^	0.86 ^1a^	0.57 ^1a^	0.34	−0.29 ^1a^	−1.14 ^1ab^	−0.29 ^1ab^	0.38	−1.29 ^1a^	−1.14 ^1ab^	−2.14 ^1ab^	0.35
Casings	L*	45.57 ^1a^	44.57 ^1a^	45.71 ^1a^	0.84	25.57 ^1b^	23.43 ^1b^	23.86 ^1b^	0.59	16.43 ^1c^	16.29 ^1b^	18.43 ^1bc^	0.97
	a*	45.29 ^1a^	43.57 ^1a^	43.57 ^1a^	0.45	38.86 ^1b^	36.14 ^12b^	34.19 ^2b^	0.57	26.57 ^1c^	24.29 ^1c^	23.43 ^1c^	0.74
	b*	19.57 ^1a^	17.00 ^12ab^	14.71 ^1a^	0.61	26.71 ^1b^	22.71 ^12a^	18.71 ^2a^	0.95	16.57 ^1a^	13.43 ^1b^	13.86 ^1a^	0.76

HF—25% of fat; MF—17.5% of fat; LF—10% of fat; SEM—standard error of mean. ^1–3^ Mean values in the same row (corresponding to the same day of ripening) followed by a different number differ significantly (*p* < 0.01).^a–c^ Mean values in the same row (corresponding to the same batch) followed by a different letter differ significantly (*p* < 0.01).

**Table 2:** Changes in geometrical, physicochemical, oxidative, textural and colour parameters of *tea* sausage produced with reduced fat content in the last fourteen days of ripening.

Processing Time (Days)													
		21			SEM	28			SEM	35			SEM
		HF	MF	LF		HF	MF	LF		HF	MF	LF	
Shrinkage (%)		24.45 ^1c^	32.27 ^2b^	40.37 ^3c^	2.31	28.00 ^1d^	37.18 ^2c^	42.45 ^3cd^	2.12	29.87 ^1d^	39.41 ^2c^	44.41 ^3d^	2.14
Chemical parameters													
pH		5.01 ^1c^	5.11 ^2d^	5.27 ^3d^	0.04	4.95 ^1b^	5.05 ^2b^	5.19 ^3e^	0.03	5.08 ^1d^	5.15 ^2e^	5.21 ^3f^	0.02
a_w_		0.908 ^1d^	0.890 ^2d^	0.866 ^3d^	0.01	0.890 ^1e^	0.872 ^2e^	0.846 ^3e^	0.01	0.875 ^1f^	0.824 ^2f^	0.807 ^3f^	0.01
Protein (% DM)		34.21 ^1a^	39.12 ^2a^	44.73 ^3a^	1.52	32.90 ^1a^	40.05 ^2a^	46.05 ^3a^	1.90	34.18 ^1a^	40.33 ^2a^	45.22 ^3a^	1.60
Fat (% DM)		55.52 ^1bc^	50.92 ^2bc^	44.34 ^3ab^	1.62	59.15 ^1a^	49.58 ^2cd^	44.31 ^3ab^	2.18	57.22 ^1b^	48.38 ^2d^	44.72 ^3a^	1.86
Moisture		40.58 ^1d^	37.67 ^2d^	35.18 ^3d^	0.78	36.28 ^1e^	34.43 ^2e^	32.70 ^3e^	0.52	33.60 ^1f^	30.76 ^2f^	29.72 ^3f^	0.58
Ash		4.12 ^1c^	4.72 ^2d^	5.28 ^3d^	0.17	4.06 ^1c^	4.86 ^2d^	5.67 ^3e^	0.23	4.54 ^1d^	5.34 ^2f^	5.84 ^3e^	0.19
Weight loss (%)		25.42 ^1c^	35.92 ^2c^	41.30 ^3c^	2.34	28.23 ^1d^	38.58 ^2d^	44.43 ^3d^	2.37	30.56 ^1e^	40.98 ^2e^	46.98 ^3e^	2.40
Lipid oxidation													
TBARS (mg MAL/kg)		0.05 ^1ac^	0.07 ^12b^	0.08 ^2b^	0.00	0.08 ^1bc^	0.06 ^1b^	0.08 ^1b^	0.02	0.07 ^1bc^	0.08 ^1b^	0.07 ^1ab^	0.00
Acid value (mg KOH/g)		3.71 ^1c^	3.63 ^1c^	4.76 ^2d^	0.18	5.84 ^1d^	5.58 ^2d^	7.65 ^3e^	0.32	6.23 ^1e^	6.72 ^2f^	7.62 ^e^	0.20
Peroxide value (meq/kg)		2.35 ^1c^	1.65 ^2c^	0.95 ^3cd^	0.20	2.63 ^1d^	1.63 ^2c^	1.05 ^3de^	0.23	2.34 ^1c^	1.93 ^2d^	1.08 ^3e^	0.19
TPA test													
Hardness (*n*)		34.30 ^1d^	48.12 ^2d^	63.67 ^3d^	2.48	45.69 ^1e^	58.59 ^2e^	74.26 ^3d^	2.55	57.12 ^1f^	70.15 ^2f^	90.64 ^3e^	3.09
Springiness		0.67 ^1ab^	0.65 ^1ac^	0.56 ^2c^	0.01	0.58 ^1ac^	0.60 ^1a^	0.55 ^1c^	0.01	0.61 ^12a^	0.63 ^1a^	0.55 ^2c^	0.01
Cohesiveness		0.46 ^1ac^	0.44 ^1a^	0.48 ^1ac^	0.01	0.40 ^1a^	0.41 ^1ac^	0.46 ^2a^	0.01	0.37 ^1ad^	0.37 ^1c^	0.44 ^2a^	0.01
Gumminess (*n*)		17.21 ^1d^	26.55 ^2d^	39.64 ^3d^	1.85	25.42 ^1e^	32.33 ^2e^	50.00 ^3e^	2.08	30.75 ^1f^	40.52 ^2f^	61.14 ^3f^	2.59
Chewiness (*n*)		12.91 ^1c^	20.56 ^2d^	31.71 ^3d^	1.57	18.65 ^1d^	28.78 ^2e^	37.27 ^3e^	1.54	25.59 ^1e^	33.79 ^2f^	44.33 ^3f^	1.72
Resilience		0.11 ^1c^	0.12 ^1b^	0.12 ^1c^	0.00	0.08 ^1abd^	0.09 ^1acd^	0.13 ^2c^	0.00	0.07 ^1ad^	0.07 ^1d^	0.12 ^2c^	0.00
Colour													
Meat	L*	44.14 ^1b^	42.86 ^1b^	40.71 ^1a^	0.89	45.57 ^1ab^	44.29 ^1b^	44.86 ^1a^	0.71	44.57 ^1ab^	45.71 ^1ab^	45.86 ^1a^	0.64
	a*	32.86 ^1b^	33.14 ^1b^	32.14 ^1b^	0.46	36.86 ^1ab^	36.57 ^1ab^	34.14 ^1bc^	0.45	39.43 ^1a^	36.57 ^1ab^	37.43 ^1c^	0.57
	b*	12.86 ^1a^	12.14 ^1ab^	14.14 ^1ab^	0.51	15.43 ^1a^	15.71 ^1ab^	13.57 ^1ab^	0.41	12.29 ^1a^	11.71 ^1b^	11.57 ^1b^	0.25
Fat	L*	75.14 ^1ab^	75.29 ^1a^	72.57 ^1b^	0.97	78.29 ^1ab^	78.14 ^1ab^	76.29 ^1ab^	0.67	77.29 ^1ab^	78.86 ^1ab^	76.71 ^1ab^	0.74
	a*	11.14 ^1ab^	9.14 ^1a^	10.57 ^1a^	0.61	9.29 ^1ab^	8.14 ^1ab^	9.86 ^1a^	0.36	6.86 ^1b^	4.71 ^1b^	5.14 ^1c^	0.38
	b*	−1.29 ^1a^	−2.00 ^1b^	−2.71 ^1ab^	0.46	−1.86 ^1a^	−2.43 ^1b^	−3.57 ^1b^	0.31	−1.14 ^1a^	−1.29 ^1ab^	−1.86 ^1ab^	0.39
Casing	L*	16.00 ^1c^	17.71 ^1b^	15.57 ^1c^	0.79	16.57 ^1c^	17.57 ^1b^	16.14 ^1c^	0.54	15.86 ^1c^	16.00 ^1b^	17.71 ^1bc^	0.74
	a*	28.86 ^1cd^	25.57 ^1c^	23.86 ^1c^	0.89	32.57 ^1d^	29.57 ^1bc^	28.57 ^1bc^	1.38	30.71 ^1cd^	27.43 ^1c^	25.00 ^1bc^	0.88
	b*	18.29 ^1a^	18.14 ^1ab^	14.57 ^1a^	0.77	17.14 ^1a^	17.43 ^1ab^	14.43 ^1a^	0.76	17.43 ^1a^	18.86 ^1ab^	15.57 ^1a^	0.72

HF—25% of fat; MF—17.5% of fat; LF—10% of fat; SEM—standard error of mean. ^1−3^ Mean values in the same row (corresponding to the same day of ripening) followed by a different number differ significantly (*p* < 0.01). ^a–f^ Mean values in the same row (corresponding to the same batch) followed by a different letter differ significantly (*p* < 0.01).

**Table 3:** Sensory evaluation of *tea* sausage formulated with different levels of fat ripened for 35 and 28 days (mean ± standard deviation).

	35 Day			28 Day		
Attributes	HF (Control)	MF	LF	HF	MF	LF
Colour	4.50 ± 0.67 ^a^	3.75 ± 0.79 ^ab^	3.33 ± 0.49 ^b^	3.83 ± 0.58 ^ab^	4.42 ± 0.79 ^a^	3.58 ± 0.51 ^ab^
Aroma	4.58 ± 0.45 ^a^	4.33 ± 0.89 ^ab^	3.33 ± 0.52 ^b^	4.08 ± 0.90 ^ab^	4.50 ± 0.67 ^ac^	3.67 ± 0.49 ^bc^
Taste	4.67 ± 0.49 ^a^	3.92 ± 0.90 ^ab^	3.42 ± 0.67 ^b^	4.00 ± 0.43 ^ab^	4.25 ± 0.96 ^ab^	3.75 ± 0.45 ^ab^
Consistency	3.96 ± 0.67 ^a^	4.08 ± 0.79 ^a^	3.83 ± 0.83 ^a^	3.67 ± 0.49 ^a^	4.17 ± 0.71 ^a^	3.92 ± 0.79 ^a^
Overall acceptability	4.67 ± 0.49 ^a^	3.83 ± 0.71 ^b^	3.08 ± 0.29 ^b^	3.75 ± 0.62 ^b^	4.75 ± 0.45 ^a^	3.42 ± 0.51 ^b^

HF—25% of fat; MF—17.5% of fat; LF—10% of fat. Values with different lowercase letters (a–c) in the same row differ significantly (*p* < 0.01).

**Table 4:** Oral processing parameters of tea sausage produced with reduced fat content (mean ± standard deviation).

	Number of Chews	Consumption Time of One Bite (s)	Chewing Rate (chew/s)	Average Bite Size (g)	Eating Rate (g/s)	Fat Intake Rate (fat/s)
HF-35	28.88 ± 3.5 ^a^	17.21 ± 1.8 ^a^	1.68 ± 0.06 ^a^	7.12 ± 0.55 ^a^	0.42 ± 0.04 ^a^	0.15 ± 0.01 ^a^
MF-28	29.67 ± 5.1 ^a^	17.46 ± 2.7 ^a^	1.69 ± 0.06 ^a^	7.04 ± 0.74 ^a^	0.41 ± 0.02 ^ab^	0.14 ± 0.01 ^b^
LF-28	32.75 ± 7.0 ^a^	20.96 ± 4.5 ^b^	1.56 ± 0.08 ^b^	7.46 ± 0.69 ^a^	0.37 ± 0.07 ^b^	0.12 ± 0.02 ^c^

HF—35–25% of fat and ripened for 35 days; MF—28–17.5% of fat and ripened for 28 days; LF—28–10% of fat and ripened for 28 days. ^a^^–c^ Mean values in the same column followed by different letter (a–c) differ significantly (*p* < 0.01).

## Data Availability

The data used to support the findings of this study are included within the article.
